# Comparison of Two X-ray Analyses for Estimating the Prognosis of Eruption of Impacted Mandibular Third Molars

**DOI:** 10.3390/diagnostics14100999

**Published:** 2024-05-11

**Authors:** Petya G. Hadzhigeorgieva-Kanazirska, Nikolay D. Kanazirski, Iliyana L. Stoeva

**Affiliations:** 1 Department of Diagnostic Imaging, Dental Allergology and Physiotherapy, Faculty of Dental Medicine, Medical University-Plovdiv, 4000 Plovdiv, Bulgaria; iliyana.stoeva@mu-plovdiv.bg; 2 Department of Oral Surgery, Faculty of Dental Medicine, Medical University-Plovdiv, 4000 Plovdiv, Bulgaria; nikolay.kanazirski@mu-plovdiv.bg

**Keywords:** CBCT, impacted mandibular third molars, retromolar eruption spaces, mesiodistal inclination

## Abstract

The objective of this study was to compare the results of the measurements made using two methods for determining the retromolar eruption spaces and the mesiodistal inclinations of impacted mandibular third molars. These are the main parameters based on which the eruption of these teeth can be predicted. A Sirona GALILEOS Compact/Comfort CBCT scanner was used for the study. A total of 127 patients were included in the study. We made the measurements using our integrated method and the standard method used in the dental practice for determining the eruption space and the mesiodistal inclination of these teeth, and then we compared the results. The mean difference between the two methods for estimating the retromolar space deficiency on the left was 1.70 mm and standard deviation (SD) 2.95; mean error of the mean was 0.29; and Student *t*-test (paired *t*-test) = 5.86, significant level of the correlation was 0.001, <0.05. Regarding the teeth on the right, it was mean 1.59 mm and standard deviation (SD) 2.98; mean error of the mean was 0.31. The *t*-test performed found a statistically significant difference between the methods in determining the retromolar eruption spaces (*t*-test (paired *t*-test) = 5.13; significant level of the correlation 0.001; *p* < 0.05). The mean difference (in degrees) between the measurements of the inclinations of the teeth on the left using the two methods was 3, 50°; SD = 7.25; mean error of the mean = 1.81; *t*-test = 2.481; significant level of the correlation 0.025; and *p* > 0.05. As for the teeth on the right, it was 2.41°, SD = 9.57, mean error of the mean = 2.39, *t*-test 0.175, significant level of the correlation = 0.863, and >0.05. No statistically significant difference was found between the two methods in measuring the inclinations of impacted third molars. The conclusion of our study is that the determination of the mesiodistal inclination of the teeth and the available eruption space using the method developed by us is more accurate compared to the standard method, because constant points and planes are used. This method allows for predicting the eruption of impacted mandibular third molars.

## 1. Introduction

Imaging methods are essential for the diagnosis and treatment of impacted teeth. The optimal radiographic imaging provides information not only about the presence of impacted teeth but also about their position relative to the adjacent tissues and teeth.

Regarding the impacted mandibular third molars, there are a number of signs by which their topographic position can be determined. Determining the retromolar spaces and their mesiodistal inclinations contributes to the prognosis of teeth eruption at an earlier age. To this end, panoramic radiography is of good diagnostic value. According to many studies, of all the conventional radiographic techniques used to assess the mandibular third molars, this method provides the most accurate estimate of their position in the jaw [1,2,3,4,5,6]. Dudhia R. et al. found that panoramic radiographs are routinely used in dental practice to diagnose impacted third molars, but in the different measurements it is important to consider the projection distortions and errors inherent in the method [3].

When planning the surgical procedure, in addition to the mesiodistal inclinations, the buccolingual inclinations of the impacted mandibular third molars should be determined. The position, distance, and possible contact between the teeth and the neurovascular bundle, in the mandibular canal, respectively, shall be specified in order to prevent complications during surgery. According to Yuxue Chen et al., the buccolingual inclinations and the actual 3D relationship between the impacted third molars and the mandibular canal cannot be visualized by 2D techniques [7]. Orthopantomography and intraoral radiography are informative in cases where there is no overlap between the mandibular canal and the roots of the impacted teeth. In cases of projection overlap of the two structures, the use of cone beam computed tomography (CBCT) is recommended. This method allows adequate determination of the distance, the buccolingual position of the teeth relative to the neurovascular bundle, and the possible contact between the teeth [8,9,10,11,12,13,14].

Superposition of the anatomical structures, magnification of the image which affects the accuracy of the measurements, projection distortion, and blurring of the image in conventional methods have been confirmed by many authors [1,15,16]. Other authors confirm the accuracy of CBCT measurements [7,17,18].

Determining the available retromolar eruption space is essential for the prognosis of the eruption of the mandibular third molars at an earlier age. Hattab, F.N. et al. argue that the most significant variable associated with third molar impaction is inadequate space. According to the authors, the space behind the second molar is reduced in 90% of cases with third molar impaction [19]. To reach this conclusion, they measured the retromolar space/mesiodistal crown width ratio of the impacted teeth on panoramic radiographs. They estimated the retromolar space by measuring the distance between the most convex point on the distal surface of the crown of the second molar and the junction of the anterior edge of the ramus with the body of the mandible. The mesiodistal crown width is the greatest distance between the medial and distal surfaces of the tooth.

Another method for measurement of the retromolar space is the method of Ganss [20]. The authors authors measure the distant between the tangent to the distal surface of the second molar the anterior edge the of the ramus of the mandible. The mesiodistal crown width is the greatest distance between the medial and distal surfaces of the tooth [20]. These measurements are standard in practice for determining the retromolar eruption space [19,20,21]. The disadvantage of these analyses is that the alveolar bone above the crown of the impacted teeth, which is often an obstacle to their eruption, is not determined. Other authors conclude that the eruption space is determined by the size of the alveolar bone of the body of the mandible [22].

Another significant variable for eruption or impaction is the third molar mesiodistal inclination. It has been proven that the greater the inclination, the greater the probability of impaction. The method most commonly used in practice for determining the mesiodistal inclination of the third molars is measuring the angulation between the long axes of the second and third molars. The long axis is determined by drawing a line between the midpoint of the occlusal surfaces and the bifurcations [3,19]. The disadvantage of the standard method of determining the inclination of impacted third molars relative to adjacent second molars is that these teeth are not a reference plane. They also change their inclination and could be missing [23,24].

Haavikko et al. made measurements on panoramic radiographs (OPGs) and found that the initial angulation of the mandibular third molars (at approximately 13 years of age) could affect their eruption. They found that when the long axes of the second and third molars at this age are parallel or the angle between them is less than 10°, most third molars will erupt (at about 19.5 years of age). According to the authors, with an initial angle of 10°–20°, the probability of impaction of the third molars is 50%. With an initial angle of over 20°, the probability of impaction is 75% [25].

## 2. Objective

The objective of our study was to compare the results of the measurements made using two methods for determining the retromolar eruption spaces and the mesiodistal inclinations of impacted mandibular third molars.

## 3. Material and Methods

### 3.1. Patient Selection

The Ethical Committee of Medical University, Plovdiv, Bulgaria, approved the present study (ethic code: P-3/08.06.2017). Patients were enrolled after informed consent was obtained, and the protocol conformed to the ethical guidelines of the 1975 Declaration of Helsinki.

The patients studied retrospectively totaled 127, aged 17 to 30 years. They were selected from the archive of the Faculty of Dental Medicine, at the Medical University-Plovdiv, Department of “Image diagnostics, dental allergy and physiotherapy”.

Inclusion criteria in the study were the following: patients with permanent dentition and stages of root development of the third molars according to the Demirjian scale. In Stage F, root length is at least as great as crown length and roots have funnel-shaped endings. In Stage G, root walls are parallel, but apices remain open. In Stage H, apical ends of the roots are completely closed, and the periodontal membrane has a uniform width around the root [26].

Exclusion criteria were the following: missing molars of the lower jaw, severe jaw atrophy, and systematic diseases affecting the bone structure.

### 3.2. Study Protocol

The patients were examined with Sirona GALILEOS Compact/Comfort (Charlotte, NC, USA), field of view 15 × 15 × 15 sm^3^, isotropic voxel size 0.15 mm, and exposure time 14 s. Voltage and amperage are determined according to the age and physique of the patient. They range from 90–120 kV and 21–42 mA, respectively. The effective dose also varies between 29 Sv and 54 Sv, at 21 mA and 42 mA, respectively.

### 3.3. Study Design

To verify the exactness of our measurements, we initiated a study in which the values of the measurements taken on the X-ray image were compared with those of the real object. For this purpose, 10 cadaver mandibulae taken from the “Department of Anatomy, Histology and Embryology”, Medical University-Plovdiv, were used. Before being scanned, certain anatomical points were determined on the mandibulae with radiopaque markers. The distances between them were measured with a digital caliper. One of the performed measurements related to the present study is the length of the vertical line drawn from the intersection of the ramus and the alveolar crest to the lower edge of the mandible. These measurements were made in mm on the cadaveric mandibulae and on the reconstructed panoramic image obtained after scanning. The measured values were compared statistically.

Some of the patients in this study were assigned a conventional panoramic X-ray before the 3D scan and subsequently CBCT for other pathological processes. In these cases, the measurements of both studies were performed to confirm the accuracy of the measurements on the panoramic reconstructed image after the patients’ scans. From the point of view of radiation protection, it was not justified to perform an additional conventional panoramic X-ray on all patients. The images and measurements were performed by a dental imaging diagnostic specialist and verified by orthodontics and oral surgery specialists.

Based on the literature data available to us, as a result of our study we developed an integrated method for determining the retromolar space and the mesiodistal inclination of impacted mandibular third molars. The measurements were taken on the reconstructed panoramic images after CBCT scanning of the patients. The panoramic curveline reconstructions were obtained using Galileos viewer 1.9. (Charlotte, NC, USA) and passed through the furcation area of the teeth of the lower jaw. Reference points and lines according to the method of Chadina T. V. et al. and the method of Ganss were used [20,22].

#### 3.3.1. Methods for Estimating Retromolar Spaces

##### Integrated Method (Method A)

On the reconstructed panoramic image obtained after scanning, we determine the following points (Table 1): T—temporal point—the lowest point of the articular tubercle of the temporal bone; M—mandibular point—the point of intersection of the tangents to the lower edge of the body of the mandible; and Ang—angular point—the angular point of intersection of the tangents to the lower edge of the body and the ascending ramus of the mandible.

From the line connecting T–T (defined as main horizontal plane), we construct the middle vertical line or the line of the esthetic center—from the midpoint of the line T–T to point M. The point of intersection of the middle vertical line with the upper end of the alveolar process of the mandible is point Al. The distance between the points Al and M is plotted above point Al on the middle vertical line as point M1.

**Figure 1:**
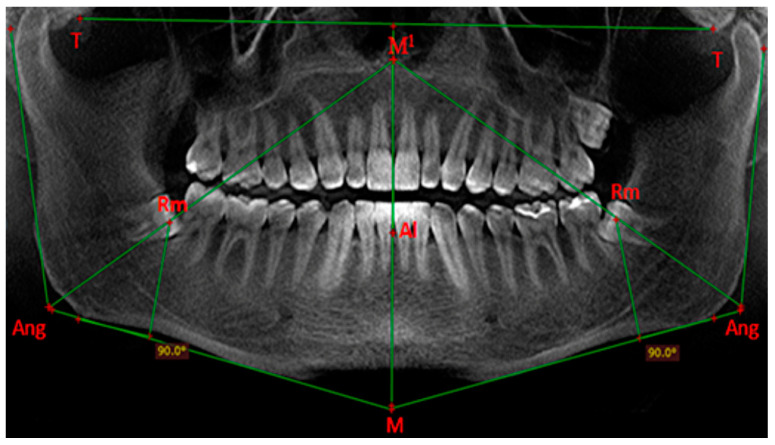
Main points and lines that we draw on the reconstructed panoramic image. Adapted from Petya G. Kanazirska, Georgi Y. Yordanov, Irina A. Angelova, Nikolai D. Kanazirski (2017).

The angular point Ang is connected to point M1, and the point of intersection of the obtained line with the ridge of the alveolar process of the mandible determines the position of the retromolar point—Rm. From the retromolar point, we draw a perpendicular to the tangent to the lower edge of the body of the mandible. We define this line as the vertical anatomical junction of the body with the ascending ramus of the mandible or the “stress-axis” line (Figure 1).

**Figure 2:**
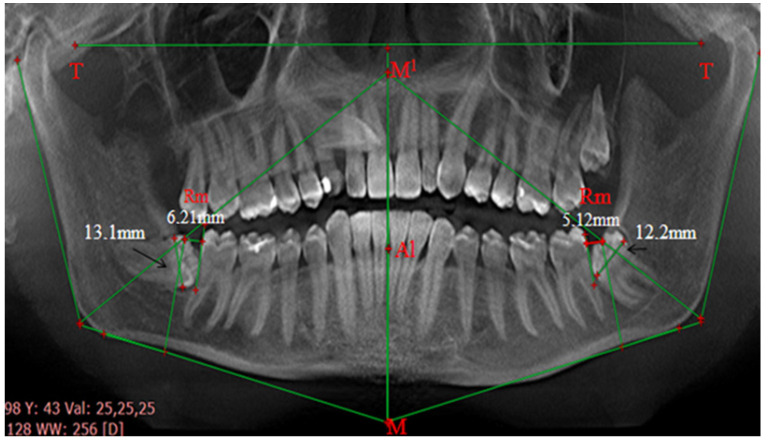
Measurement of the retromolar spaces and the mesiodistal dimensions of the crowns using the integrated method. Reprinted from Petya G. Kanazirska, Georgi Y. Yordanov, Irina A. Angelova, Nikolai D. Kanazirski (2017).

After determining this “stress axis” line or the conditional vertical line, we determine the retromolar space. This is the distance (in mm) from the most prominent point of the second molar to the vertical line. Then we compare the measured retromolar space with the mediodistal dimension of the impacted tooth (the distance between the most protruding mesial and distal points of the crown) (Figure 2).

##### Standard Method (Method B)

**Figure 3:**
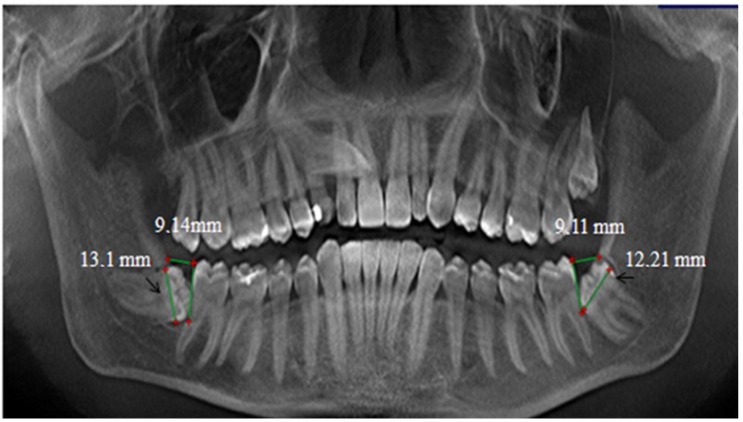
Measurement of the retromolar spaces and the mesiodistal dimensions of the crowns (indicated by the arrows) using the standard method. Adapted from Petya G. Kanazirska, Georgi Y. Yordanov, Irina A. Angelova, Nikolai D. Kanazirski (2017).

In this study, the retromolar space/mesiodistal crown width ratio of impacted teeth was estimated using the standard method, which is most commonly used in practice. We measured the distance between the most convex point on the distal surface of the crown of the second molar and the junction of the anterior edge of the ascending ramus with the body of the mandible. The mesiodistal crown width is the greatest distance between the medial and distal surfaces of the tooth (Figure 3).

#### 3.3.2. Methods for Determining the Mesiodistal Inclinations of Impacted Mandibular Third Molars

##### Integrated Method (Method A)

**Figure 4:**
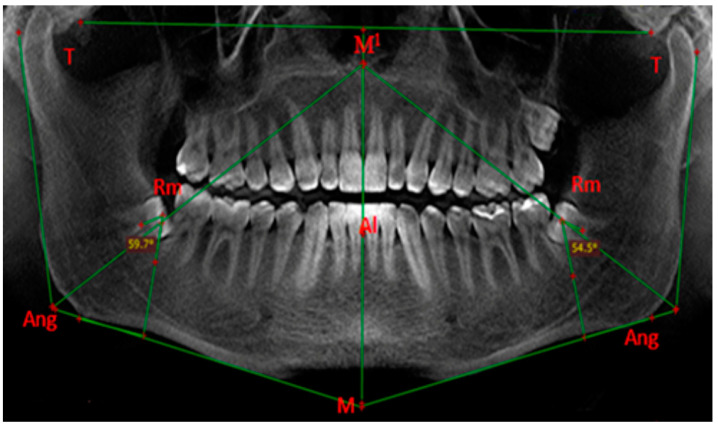
Measurement of the mesiodistal inclinations of impacted third molars in degrees relative to the vertical junction of the body with the ascending ramus of the mandible. Reprinted from Petya G. Kanazirska, Georgi Y. Yordanov, Irina A. Angelova, Nikolai D. Kanazirski (2017).

After finding the retromolar point as described, we draw the longitudinal axes of the mandibular third molars. These are the lines passing through the midpoints of the occlusal planes and the midpoints of the bifurcations. Then we measure the angles between the longitudinal axes and the perpendicular lines drawn from the retromolar point to the tangent to the lower edge of the mandible (Figure 4). When the point of intersection of the perpendicular line and the longitudinal axis of the tooth is upwards (in the direction of the masticatory surface of the impacted tooth), we denote the degree by the symbol “+”. When the point of intersection of the perpendicular line and the longitudinal axis of the tooth is downwards (in the direction of the bifurcation of the impacted tooth), we denote the degree by the symbol “−”. Then we determine the mesiodistal inclination of the teeth according to the Winter’s classification:

1. vertical position, from +10° to −10°;
2. mesial inclination, from +10° to +70°;
3. distal inclination, from −10° to −70°;
4. horizontal position, over ±70°.

##### Standard Method (Method B)

**Figure 5:**
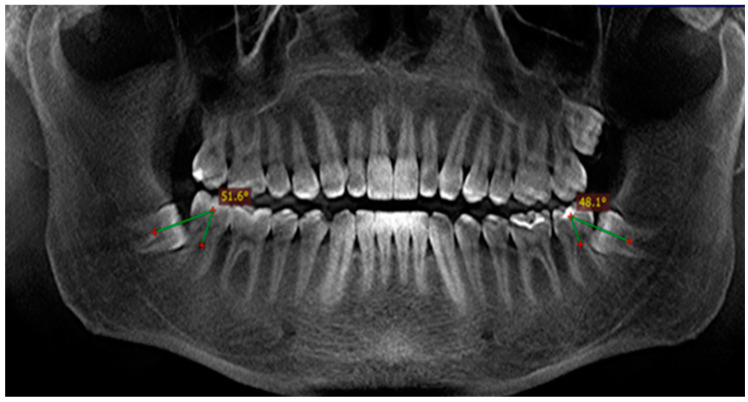
Measurement of the mesiodistal inclinations of the impacted third molars in degrees relative to the longitudinal axes of the adjacent second molars (standard method). Adapted from Petya G. Kanazirska, Georgi Y. Yordanov, Irina A. Angelova, Nikolai D. Kanazirski (2017).

The mesiodistal inclination of the impacted third molars in our study was also determined using the standard method (Figure 5). The longitudinal axes of the impacted third molars and the adjacent second molars were drawn, after which we measured the angles between them in degrees. Based on the measured angles, we again determined the mesiodistal inclination of the teeth according to the Winter’s classification.

##### Surgical Technique

In patients with partially impacted third molars surgical intervention was performed to aid their eruption. Their mesiodistal inclination was up to 10°, and 75–80% of the mesiodistal diameter of these teeth was in front of the “stress-axis” line. The occlusal plane of these teeth was above the cervical region of the adjacent tooth. For the surgical intervention we used a high-energy Er:YAG laser, Litetouch, (Light Instruments Ltd.,Yokneam, Israel), with a wavelength of 2940 nm, for circumcision of the soft tissues and osteotomy of the underlying bone. The advantages of the method are reduced bleeding and pain during the procedure. Ablation and decontamination of the area is performed, which is a prerequisite for a reduced inflammatory process and swelling in the postoperative period.

The soft tissue circumcision protocol was as follows: release incision flap contact, laser energy 200 mj, pulse frequency 35 Hz, tip diameter × length 0.4 × 17 mm, and water spray level 5–6.

The osteotomies were made on the bone overlying the crown and distal to the cervical region of the wisdom teeth. The work protocol was as follows: bone remodeling non-contact, laser energy 300 mj, pulse frequency 25 Hz, tip diameter × length 1.3 × 19 mm, and water spray level 8.

Statistical analysis was done with SPSS Statistics software (Version 16.0: SPSS Inc., Chicago, IL, USA) и MS Office Excel 2010). The sensitivity and specificity assessment was performed.

## 4. Results

The results of the measurements made according to the integrated method (method A) for determining the retromolar eruption space are as follows: in 22 teeth (11.3% ± 2.3) there was enough space in the jaw for eruption, and retromolar space deficiency was found in 173 teeth (88.7% ± 2.3). According to the measurements made by the standard method (method B), the teeth with enough eruption space totaled 29 (14.9% ± 2.5). Retromolar space deficiency was found in 166 teeth (85.1% ± 2.5).

In this study, we compared the mean (in mm) of the eruption spaces and the widest parts of the crowns of all teeth using both methods. Then we found the mean values of retromolar space deficiency in mm. According to the method A, the eruption space deficiency for the teeth on the left was mean = −4.34 mm, standard deviation (SD) = 3.55, and mean error of the mean = 0.35. According to the method B it was mean = −2.64 mm, SD = 2.85, and mean error of the mean = 0.28. There is a correlation between the two methods and significance, r = 0.595, *p* = 0.000, and *p* < 0.05. For the teeth on the right, according to the method A, the eruption space deficiency was mean = −4.40 mm, SD = 3.79, and mean error of the mean = 0.39, and in method B—mean = −2.81 mm, SD = 2.90, and mean error of the mean = 0.30. There is a correlation between the two methods and statistical significance, r = 0.632, *p* = 0.000, and *p* < 0.05 (Table 2).

The mean difference between the two methods for estimating the retromolar space deficiency on the left was 1.70 mm, SD = 2.95, mean error of the mean = 0.29, Student *t*-test (paired *t*-test) = 5.86, *p*-value significance level, = 0.001, and *p* < 0.05. Regarding the teeth on the right, it was mean 1.59 mm, SD = 2.98, mean error of the mean = 0.31, *t*-test = 5.13, *p* = 0.001, and *p* < 0.05. The *t*-test performed found a statistically significant difference between the methods in determining the retromolar eruption spaces (Table 3).

The most common inclination of the mandibular third molars with delayed eruption, determined relative to the conditional vertical (Method A), was mesial inclination in 120 teeth (61.5% ± 3.5). Second in frequency was vertical position—34 teeth (17.4% ± 2.7)—followed by distal inclination in 21 teeth (10.8% ± 2.2). The least common teeth were those positioned horizontally—20 teeth, 10.3% ± 2.2.

The sequence of the frequency of mesiodistal inclination of mandibular third molars as determined relative to the longitudinal axes of the adjacent second molars (Method B) was different. The teeth with medial inclination were the most frequent—126 (64.6% ± 3.4). Second in frequency was distal inclination—28 teeth (14.4% ± 2.5)—followed by horizontally positioned teeth, 23 (11.8% ± 2.3). The teeth with vertical positioning were the least frequent—18 (9.2% ± 2.1).

In 16 teeth on the left and 16 on the right, different inclinations were determined using the two methods. The mean of the inclinations in degrees of these teeth on the left by the method A was 14.46°, SD = 27.37, and mean error of the mean = 6.84. According to the method B mean was 17.96°, SD = 30.38, and mean error of the mean = 7.59. For teeth on the right the values respectively were the following: in method A, mean = 7.54°, SD = 18.20, and mean error of the mean = 4.55, in method B, 9.96°, 23.95, and 5.98, respectively. There is a correlation between the two methods, r = 0.933 with a statistically significant *p*-value—*p* = 0.000; *p* < 0.05. The mean difference (in degrees) between the measurements of the inclinations of the teeth on the left using the two methods was 3.50°, SD = 7.25, mean error of the mean = 1.81, *t*-test = 2.481, *p* = 0.025, and *p* > 0.05. As for the teeth on the right, it was mean = 2.41°, SD = 9.57, mean error of the mean = 2.39, *t*-test = 0.175, *p* –value significance level = 0.863, and *p* > 0.05. No statistically significant difference was found between the two methods in measuring the inclinations of impacted third molars (Table 4).

To verify the exactness of our measurements, we initiated a study in which the values of the measurements taken on the X-ray image were compared with those of the real object. The length of the vertical line drawn from the intersection of the ramus and the alveolar crest to the lower edge of the mandible was measured with a digital caliper in mm on the cadaveric mandibulae and after that on the reconstructed panoramic image obtained after scanning. After performing a paired samples statistics test, it was found that the average value of the perpendicular line drawn from the retromolar point to the edge of the lower jaw on the right, measured with a caliper, was a mean of 16.02 mm and SD of 5.58. The measured values of the same perpendicular line of the panoramic reconstruction were a mean of 16.89 mm and SD of 4.14. In the measurements on the left, the values were, respectively, the following: with a caliper, mean 16.53 mm and SD 5.63, and on the X-ray examination mean 17.64 mm and SD 5.42. The *t*-test performed found a statistically insignificant difference between the methods in the measurements between the real object and the X-ray method. On the left the mean value was −0.88 and there was a significant correlation level of 0.305, >0.05, and on the right the mean value was −1.11 and there was a significant correlation level 0.213, >0.05.

After the analysis was performed based on the method proposed by us, it was found that in 12 impacted third molars (9.5% ± 2.1) 75–80% of the mesiodistal diameter of these teeth was in front of the conditional vertical, and their mesiodistal inclination was up to 10°. These teeth were partially impacted in the bone, and alveolar bone could be seen above the distal part of their crowns, which coincided with the retromolar point. Circumcision and osteotomy were performed in these teeth using an Er-YAG laser, and they reached the occlusal plane.

## 5. Discussion

The patients’ ages in our study ranged from 17 to 30 years. In selecting the age group of the patients, we based our selection on the statement of Faiez N. et al. that the jawbones continue to grow until the age of 17. They argue that 20 years is the age at which it can be determined whether the conditions for a normal eruption are present [19]. Kruger E. et al. concluded that by the age of 26, impacted third molars undergo favorable changes in their position leading to eruption [27].

In our study, the mean of the retromolar space, with enough space for eruption of the third molars (according method A), was 8.96 mm, SD = 2.62, and mean error of the mean = 0.17. The results of other authors’ measurements (according to the standard method) show larger average values of the retromolar space required for tooth eruption. According to Ganss et al., this space is 15 mm [20]. According to other authors, the mean retromolar space, in which sufficient space for eruption of third molars is established, is 10.5 mm, which is close to the value of our measurements made by method B of mean 9.92 mm, SD = 3.78, and S = 0.19 [19,28].

Student’s *t*-test (paired *t*-test) performed found a statistically significant difference between the two methods in determining the retromolar eruption spaces. The analysis of the results of the measurements made shows a lower mean of the retromolar space when using the method developed by us compared to the standard method. This is due to the fact that occlusal surfaces in hard tissue tooth impaction are always covered (partially or completely) with bone. The line drawn from point M1 to the angular point Ang intersects this bone at the alveolar ridge. The retromolar point determined using the method developed by us is also there, and this is the advantage of the method. In the standard method, the retromolar point is the point of intersection of the anterior edge of the ascending ramus and the body of the mandible. This method does not consider the alveolar bone over the occlusal surface of the teeth. However, this bone can be an obstacle to eruption. This is confirmed in the study of Chadina T. V. et al., who found that the eruption space is determined by the size of the alveolar bone of the body of the mandible [22].

The most common inclination of the mandibular third molars with delayed erup-tion, determined relative to the conditional vertical (Method A), was mesial inclination of 61.5%. Second in frequency was vertical position, 17.4%, followed by distal inclination of 10.8%. The least common teeth were those positioned horizontally—10.3%. Our results (Method A) are close to the results in the study by Dudhia et al. Teeth with a medial inclination, according to their results, are 44.8%, followed by those with a vertical inclination, 28.8%; distal inclination, 13.5%; and with the least frequency are horizontally located, 12.8% [3]. Lübbers et al. also define this as the most common the medial and vertical position of the teeth. But in their study, in frequency before the distal inclination of the teeth is the horizontal [29].

The sequence of the frequency of mesiodistal inclination of mandibular third molars as determined relative to the longitudinal axes of the adjacent second molars (Method B) was different. The teeth with medial inclination were the most frequent—64.6%. Second in frequency was distal inclination, 14.4%, followed by horizontally positioned teeth—11.8%. The teeth with vertical position were the least frequent—9.2%. These data are close to the results of other authors [30].

In 16 teeth on the left and 16 on the right, different inclinations were determined using the two methods. In six teeth on the left, we found mesial inclination using the standard method and vertical inclination using the method developed by us. In four teeth, we found distal inclination using the standard method and vertical inclination based on the measurements by the new method. Four teeth had horizontal positions relative to the second molar by the standard method and mesial inclination by the new method, respectively. By the standard method, two teeth had vertical positions, but based on the measurements used in our integrated method they had distal inclination. The difference in the inclinations of these teeth is due to the fact that they were in a borderline position between the respective inclinations.

Based on these results, we can conclude that the difference of the mean inclination of the third molars relative to the vertical (integrated method) and the mean inclination relative to the longitudinal axis of the adjacent second molars (standard method) is equal to the mean mesiodistal inclination of the adjacent second molars. The second molars are not a reference plane because they may also have mesiodistal inclination or be absent in some cases.

The differences in the degrees of inclination between the two methods can be explained by the fact that the longitudinal axes of the seventh teeth are also inclined. They are not reference planes. This statement confirms the results of the studies of Jaina S. et al. and Turkoza C. et al. [23,24]. Jaina S. et al. measured the inclinations in degrees of the third and second molars relative to a reference horizontal line passing through the anterior nasal spine (ANS) and the shadow in which the hard palate is projected [23]. Turkoza C. et al. measured the inclination of the impacted third molars and the adjacent second molars relative to the tangent to the edge of the body of the mandible. In their studies, the authors extracted the first premolars in order to see if this could be of benefit to the eruption of the third molars. They concluded that, in addition to the inclination of the third molars, the inclination of the second molars also changes [24].

The value of the analysis developed by us is that it can be easily and quickly used in the orthodontic practice of classic panoramic imaging and panoramic reconstruction. That is why we do not use 3D morphometric points. The three-dimensional reconstructions after the 3D scan were used in the teeth that have no chance of eruption to determine their position in relation to the vascular nerve bundle, their buccolingual position, and to diagnose pathological processes associated with them. This necessitates the surgical treatment plan.

The purpose of comparing method A with method B is to prove that there is a difference between the standard method and the integrated method in this study. This method allows prediction the eruption of impacted mandibular third molars, because constant points and planes are used.

We can also conclude that if 75% to 95% of the mediodistal diameter of the teeth is in front of the conditional vertical and has an angulation of +10 to −10, these teeth have a chance of eruption. Teeth in which from 26% to 74% of the mediodistal diameter of the teeth is in front of the conditional vertical and are semi-impacted. And less than 25% of the mediodistal diameter of the teeth is in front of the vertical, they are impacted.

## 6. Conclusions

Determination of the mesiodistal inclination of the teeth and the available eruption space using the method developed by us allows accurate prediction of the eruption of impacted mandibular third molars, because constant points and planes are used.

## Figures and Tables

**Table 1:** Referent points and lines.

T—temporal point	the lowest point of the articular tubercle of the temporal bone
M—mandibular point	the point of intersection of the tangents to the lower edge of the body of the mandible
Ang—angular point	the angular point of intersection of the tangents to the lower edge of the body and the ascending ramus of the mandible
Main horizontal plane	the line connecting the points T–T
Middle vertical line	the line from the midpoint of the line T–T to point M
Al—alveolar point	intersection of the middle vertical line with the upper end of the alveolar process of the mandible
M1 point	the distance between the points Al and M and plotted above point Al on the middle vertical line
Rm—retromolar point	the point of intersection of the line connecting Ang—M1 with the ridge of the alveolar process of the mandible
“Stress-axis” line	the perpendicular line from the retromolar point to the tangent to the lower edge of the body of the mandible

**Table 2:** Mean values of retromolar space deficiency in mm. *N*—total number; $\bar{X}$—arithmetic mean; SD—standard deviation; S$\bar{X}$—mean error of the mean; r—correlation confident; *p*—significant level of the correlation.

Mean Values of Retromolar Space Deficiency						
		*N*	$\bar{X}$	SD	$S\bar{X}$	r; *p*
Teeth on the left	Method A	92	−4.34	3.55	0.35	r = 0.595 *p* = 0.000; *p* < 0.05
	Method B	89	−2.64	2.85	0.28	
Teeth on the right	Method A	81	−4.40	3.79	0.39	r = 0.632 *p* = 0.000; *p* < 0.05
	Method B	77	−2.81	2.90	0.30	

**Table 3:** Mean values of the difference between Method A and Method B. $\bar{X}$—arithmetic mean; SD—standard deviation; S$\bar{X}$—mean error of the mean; CI—confidence interval; t—Student *t*-test (paired *t*-test); *p*-value—significance level.

Mean Difference between Method A and Method B						
	$\bar{X}$	SD	$S\bar{X}$	(95% CI)		t; *p*
				Lower	Upper	
Teeth on the left	1.70	2.95	0.29	1.13	2.28	t = 5.86 *p* = 0.001 *p* < 0.05
Teeth on the right	1.59	2.98	0.31	0.98	2.21	t = 5.13 *p* = 0.001 *p* < 0.05

**Table 4:** Mean difference (in degrees) between the methods. $\bar{X}$—arithmetic mean; SD—standard deviation; S$\bar{X}$—mean error of the mean; CI—confidence interval; t—Student’s *t*-test (paired *t*-test); *p*-value—significance level.

Mean Difference (in Degrees) between Method A and Method B						
	$\bar{X}$	SD	$S\bar{X}$	(95% CI)		t; *p*
				Lower	Upper	
Teeth on the left	3.50	7.25	1.81	0.63	8.36	t = 2.481 *p* = 0.065 *p* < 0.05
Teeth on the right	2.41	9.57	2.39	−4.68	5.51	t = 0.175 *p* = 0.863 *p* < 0.05

## Data Availability

The data that support the findings of this study are available from the corresponding author upon reasonable request.
